# Association of a Large Panel of Cytokine Gene Polymorphisms with Complications and Comorbidities in Type 2 Diabetes Patients

**DOI:** 10.1155/2015/605965

**Published:** 2015-05-06

**Authors:** K. F. Rodrigues, N. T. Pietrani, V. C. Sandrim, C. M. A. F. Vieira, A. P. Fernandes, A. A. Bosco, K. B. Gomes

**Affiliations:** ^1^Instituto de Ciências Biológicas, Universidade Federal de Minas Gerais, Belo Horizonte, MG, Brazil; ^2^Instituto de Biociências, Universidade Estadual Paulista Júlio de Mesquita Filho, Botucatu, SP, Brazil; ^3^Instituto de Ensino e Pesquisa, Santa Casa de Belo Horizonte, Belo Horizonte, MG, Brazil; ^4^Departamento de Análises Clínicas e Toxicológicas, Faculdade de Farmácia, Universidade Federal de Minas Gerais, Avenida Antônio Carlos, No. 6627, Pampulha, 31270-901 Belo Horizonte, MG, Brazil

## Abstract

*Aims*. The polymorphisms of pro- and anti-inflammatory cytokines may be involved in type 2 diabetes (T2D) pathogenesis and its complications. *Methods*. We investigated in 102 T2D patients the association of the cytokine polymorphisms in the TNF-*α*, IL-10, IL-6, TGF-*β*1, and IFN-*γ* genes with the T2D microvascular complications and comorbidities (hypertension, dyslipidemia, and obesity). Cytokine genotypes were determined by PCR using Cytokine Genotyping Tray kit. *Results.* Diabetic retinopathy was associated with GG genotype and G allele in TGF-*β*1 codon 25C/G polymorphism (*p* = 0.004 and *p* = 0.018) and the nephropathy was associated the lower frequency of GG genotype in IL-10 -1082G/A polymorphism (*p* = 0.049). Hypertension was associated with the CC genotype and C allele for IL-10 -592C/A polymorphism (*p* = 0.013 and *p* = 0.009) and higher frequencies of T (*p* = 0.047) and C (*p* = 0.033) alleles of the TGF-*β*1 codon 10T/C and IL-10 -819T/C polymorphisms, respectively. The TGF-*β*1 codon 10T/C polymorphism was associated with the BMI groups (*p* = 0.026): the CC genotype was more frequent in the group with BMI < 25 Kg/m^2^, while the TC genotype was more frequent in the group with BMI = 30 Kg/m^2^. *Conclusions*. Our findings suggest that TGF-*β*1 and IL-10 polymorphisms are involved in complications and comorbidities in T2D patients.

## 1. Introduction

Type 2 diabetes (T2D) is the most common form of diabetes and an increasingly prevalent metabolic disease. It is associated with microvascular and macrovascular complications and is considered one of the major causes of morbidity and mortality [1]. According to the International Diabetes Federation (IDF) [2], there were approximately 382 million people worldwide with diabetes in 2013, and this number is expected to reach more than 592 million by 2035.

In recent decades, several studies have shown the role of chronic low-grade inflammation and activation of the immune system in the pathogenesis of T2D and its complications [3–7]. However, the mechanisms by which chronic inflammation is involved in T2D are not completely clear. It has been reported that synthesis and release of pro- and anti-inflammatory cytokines such as tumor necrosis factor-*α* (TNF-*α*), interleukin- (IL-) 10, and IL-6 and growth factors such as transforming growth factor-*β*1 (TGF-*β*1) [8–13] may be involved in pathogenesis of T2D and its complications. Single nucleotide polymorphisms (SNPs) in the cytokine genes are usually located in their regulatory regions and affect the levels of their expression [14, 15]. Various studies have associated cytokine gene expression alterations with obesity, changes in insulin sensitivity, and risk of T2D [6, 7, 16].

Some works have investigated the association between the TNF-*α* -308G/A polymorphism (rs1800629) and diabetic retinopathy (DRP), nephropathy (DNP), and neuropathy (DNR) in different populations [17–24]. Only a recent Brazilian study [20] found an association between proliferative DRP and these TNF-*α* polymorphism. DNP was associated with the polymorphisms IL-10 -592C/A (rs1800872) [25] and IL-10 -819T/C (rs1800871) [26]. In addition, Paine et al. [18] associated the polymorphism IL-10 -1082G/A (rs1800896) with DRP and Kolla et al. [24] observed an association with DNR. The human TGF-*β*1 gene presents two hotspot focuses: codon 10T/C (rs1800470) and codon 25C/G (rs1800471). Both polymorphisms were associated with DRP by Beránek et al. [27], and El-Sherbini et al. [28] found an association between DNP and the codon 10T/C polymorphism. Meta-analysis conducted by Zhou et al. (2014) associated the TT genotype of TGF-*β*1 codon 10T/C polymorphism with DNP risk [29].

IL-6 gene polymorphism -174G/C (rs1800795) was associated with DNP in study conducted by Muammer et al. (2014) [30]. The few studies that evaluated the association of the polymorphism IFN-*γ* +874T/A (rs2430561) with diabetes complications found association between this polymorphism and DRP [31] and with DNR [24].

In spite of the existence of several reports examining the association of polymorphisms in various cytokine genes, much controversy remains about their role in diabetes complications. No studies to date have examined in a single population a large number of polymorphisms (TNF-*α*  -308G/A, IL-10 -1082G/A, IL-10 -819T/C, IL-10 -592C/A, TGF-*β*1 codon 10T/C, TGF-*β*1 codon 25C/G, IL-6  -174G/C, and IFN-*γ* +874T/A) and their association with the T2D complications and comorbidities.

In this study, we investigated if these polymorphisms are associated with DRP, DNP, and DNR and with comorbidities (hypertension, dyslipidemia, and obesity) in a group of Brazilian T2D patients.

## 2. Material and Methods

### 2.1. Subjects

This study was conducted with 102 Brazilian individuals, including 19 men and 83 women with clinical and laboratory diagnosis of T2D, aged from 32 to 70 years (54.99 ± 8.97 years), recruited from the Clinic of Endocrinology, Santa Casa Hospital (Belo Horizonte, Minas Gerais, Brazil) in the period from June 2012 to September 2013. T2D diagnosis was based on the American Diabetes Association (ADA) [32]. DRP was diagnosed by ophthalmoscopic examination through fundoscopic examination and slit lamp microscopic examination with present lens. DNP was defined as albumin excretion rate (AER) > 30 mg/24 h and without coexisting renal diseases from causes other than diabetes; and no DNP was defined at an AER < 30 mg/24 h, at least 2 out of 3 urine collections over a 3-month period. DNR was defined according to Diabetes Control and Complication Trial (DCCT) [33] criteria. The physical examination by neurologist and the presence of signs and symptoms (dysesthesias, paresthesias, hypersensitivity to touch, or burning pain) or absent deep tendon reflexes configured DNR presence. Two patients could not be classified according to the presence or absence of DNP due to lack of laboratory data. The determination of hypertension (systolic blood pressure ≥ 140 mmHg or diastolic blood pressure ≥ 80 mmHg or use of antihypertensive drugs) and dyslipidemia (LDL-cholesterol ≥ 100 mg/dL, HDL-cholesterol ≤ 50 mg/dL, and triglycerides ≥ 150 mg/dL or treatment of hyperlipidemia) is also in accordance with the criteria adopted by ADA [32]. Clinical data (body mass index—BMI, T2D onset) and laboratory data (fasting and postprandial glucose, HbA1c) were obtained from medical records. Subjects older than 70 years, with cancer or autoimmune disease, with current or recent infectious process, with history of cardiovascular disease, and with treatment of anti-inflammatory drugs, were excluded.

The project was approved by the Ethics Committee of the Federal University of Minas Gerais and Santa Casa Hospital. Informed consent was obtained from all the patients.

### 2.2. Genomic DNA Isolation and Genotyping

Genomic DNA was obtained from a sample of peripheral blood collected in EDTA. The Biopur Mini Spin kit (Biometrix) was used for the DNA extraction. The polymorphisms were determined using the Cytokine Genotyping Tray kit (One Lambda) which employs polymerase chain reaction-sequence specific primers (PCR-SSP), followed by electrophoresis in 2.5% agarose gel stained with GelGreen Stain (Biotium). We investigated the polymorphism TNF-*α*  -308G/A, IL-10 -1082G/A, IL-10 -819T/C, IL-10 -592C/A, TGF-*β*1 codon 10T/C, TGF-*β*1 codon 25C/G, IL-6 -174G/C, and IFN-*γ* +874T/A, which were selected due to evidence from previous studies that showed their association with the regulation in cytokines gene expression.

For association analyses of polymorphisms, patients were divided into groups according to the complication (DRP, DNP, and DNR) and comorbidity presented (hypertension, dyslipidemia, and BMI—categorized into BMI < 25 Kg/m^2^, BMI 25–30 Kg/m^2^, and BMI ≥ 30 Kg/m^2^).

### 2.3. Statistical Analysis

Deviations from Hardy-Weinberg equilibrium (HWE) were tested using an exact test (available at http://genepop.curtin.edu.au/genepop_op1.html). All of the statistical analyses were performed with Statistical Package of the Social Sciences (SPSS) version 13.0. The analysis of normality was performed by Shapiro-Wilk test. Data are presented as mean ± standard deviation (normal variables), median (interquartile range (IQR)) (no normal variables), or percentage of total (categorical variables).

Differences in the allele and genotype frequencies between the groups (DRP, DNP, DNR, hypertension, dyslipidemia, and BMI) were tested by Pearson's *χ* ^2^-test or Fisher's exact test with residuals test for three groups. For normal variables, Student's* t*-test was used to determine differences in means; for no normal variables, Kruskal-Wallis and Mann-Whitney tests were used to determine differences in medians.

IL-10 haplotype estimation was done by software Phase 2.1. We excluded haplotypes whose frequency was less than 5%. The differences in the haplotype frequencies between the groups (DRP, DNP, DNR, hypertension, dyslipidemia, and BMI) were tested by *χ* ^2^-test. A *p* value < 0.05 was considered statistically significant.

## 3. Results

The T2D patients were classified according to the complications listed above. As expected, individuals with DRP were older than those without this complication (*p* = 0.013). A slight tendency toward higher fasting glucose was observed in patients with DRP (*p* = 0.074); however, postprandial glucose level was significantly increased in this group (*p* = 0.013) when compared to individuals without DRP. This complication was significantly more frequent in patients with more than 10 years since T2D diagnosis when compared to patients diagnosed less than 5 or between 5 and 10 years earlier (*p* < 0.0001). Moreover, BMI was decreased in DRP patients (*p* < 0.0001). No significant difference was observed between the groups regarding gender, HbA1c, and the frequency of hypertension and dyslipidemia (*p* > 0.05) (Table 1).

T2D patients with DNP presented significantly increased fasting and postprandial glucose levels and HbA1c%. As seen in the DRP group, patients with nephropathy were older and the BMI was lower than the group without this complication (*p* < 0.0001 for both). Patients with T2D onset >10 years were the most frequent group presenting DNP (*p* = 0.001). No significant differences were observed for gender, hypertension, and dyslipidemia frequencies between DNP positive and negative patients, similarly to the results for the DRP group reported above (Table 2).

Regarding DNR, we observed a significant difference concerning age and gender between the groups presenting this complication or not (*p* = 0.012 and *p* = 0.003, resp.). DNR occurred more frequently in older and in female patients. The other variables were not significantly different between DNR positive and negative patients (*p* > 0.05), although a trend toward more dyslipidemic individuals was observed in the DNR group (*p* = 0.079) (Table 3).

Next, we performed an analysis of genotype and allele frequencies involving a large number of polymorphisms for cytokine genes including TNF-*α*, IL-10, TGF-*β*1, IL-6, and INF-*γ* for all complication groups. We found that all polymorphisms were under Hardy-Weinberg equilibrium (*p* > 0.05 for all).

We observed that DRP is associated with the GG genotype and G allele in TGF-*β*1 codon 25C/G polymorphism (*p* = 0.004, OR 5.865, and CI 95% 1.685–20.414 and *p* = 0.018, OR 3.387, and CI 95% 1.069–11.064). The DRP patients presented the following genotype and allele frequencies: GG 92.42%, GC 6.06%, CC 1.52%, G 95.45%, and C 4.55%, whereas patients without DRP showed the following frequencies: GG 72.22%, GC 27.78%, CC 0%, G 86.11%, and C 13.89%. In addition, DNP is associated with lower frequency of GG genotype in IL-10 -1082G/A polymorphism (*p* = 0.049, OR 2.357, and CI 95% 1.612–9.076). The frequencies observed for patients with DNP, were GG 8.75%, GA 50.00%, and AA 41.25%, and patients without DNP, GG 25.00%, GA 25.00%, and AA 50.00%. No other polymorphism was associated with DRP, DNP, or DNR (see Table 1 in Supplementary Material available online at http://dx.doi.org/10.1155/2015/605965).

Since IL-10 -1082G/A polymorphism showed association with DNP, an analysis of haplotype frequencies of IL-10 polymorphisms (-1082G/A, -819T/C, and -512C/A) was performed in the patients grouped according to the T2D complications. However, no haplotype was associated with DRP, DNP, or DNR (Table 4).

Interestingly, individuals who presented the AA genotype in IFN-*γ* +874T/A polymorphism presented lower fasting glucose levels when compared to the other genotypes: TT [median (IQR) = 146.0 (106.0) mg/dL], TA [134.0 (65.0) mg/dL], and AA [114.0 (53.5) mg/dL]; [TT × TA (*p* = 0.783); TT × AA (*p* = 0.045); TA × AA (*p* = 0.015)].

The patients also were evaluated according to the presence of comorbidities such as hypertension, dyslipidemia, and BMI categories concerning the presence of the same polymorphisms. The analysis of the genotype frequencies revealed the association of hypertension with the CC genotype and C allele for IL-10 -592C/A polymorphism (*p* = 0.013, OR 9.400, and CI 95% 1.509–58.568 and *p* = 0.009, OR 3.830, and CI 95% 1.206–12.546, resp.). The frequencies observed were patients with hypertension (CC 50.00%, CA 39.36%, AA 10.54%, C 69.68%, and A 30.32%) and patients without hypertension (CC 25.00%, CA 25.00%, AA 50.00%, C 37.50%, and A 62.50%). A higher frequency of T allele for the TGF-*β*1 codon 10T/C (*p* = 0.047, OR 2.810, and CI 95% 1.890–9.148) and C allele for the IL-10 −819T/C (*p* = 0.033, OR 0.338, and CI 95% 1.107–1.154) was also observed in patients with hypertension.

Moreover, a significant association was found between the polymorphism TGF-*β*1 codon 10T/C and BMI groups (*p* = 0.026, OR 1.963 and CI 95% 1.007–3.826 and OR 1.546 and CI 95% 1.858–2.786). The CC genotype was more frequent in the group with BMI < 25 Kg/m^2^, while the TC genotype was more frequent in the group with BMI ≥ 30 Kg/m^2^. No difference was found in the group with BMI 25–30 Kg/m^2^. The frequencies observed were BMI < 25 Kg/m^2^ (TT 26.32%, TC 42.10%, and CC 31.58%), BMI 25–30 Kg/m^2^ (TT 48.00%, TC 40.00%, and CC 12.00%), and BMI ≥ 30 Kg/m^2^ (TT 31.04%, TC 62.07%, and CC 6.89%). We also found a trend for association of the polymorphism IL-10 -592C/A and the BMI groups (*p* = 0.058). In this case, the AA genotype was less frequent in the BMI ≥ 30 Kg/m^2^ group. No other polymorphism was associated with hypertension, dyslipidemia, or BMI categories (Table 2 in Supplementary Material).

Since IL-10 -592C/A polymorphism showed an association and a tendency for association with hypertension and BMI groups, respectively, an analysis of haplotype frequencies for IL-10 polymorphisms (-1082G/A, -819T/C, and -512C/A) was performed in the patients grouped according to T2D comorbidities. For this analysis, BMI was categorized into two groups: BMI < 30 Kg/m^2^ and BMI ≥ 30 Kg/m^2^. However, no haplotype was associated with hypertension, dyslipidemia, and BMI categories (Table 4).

## 4. Discussion

This study investigated the association of cytokine gene polymorphisms in T2D patients including T2D complications DRP, DNP, and DNR, as well as comorbidities commonly observed in these patients: hypertension, dyslipidemia, and obesity.

When the patients were grouped according to T2D complications, the clinical and laboratory characteristics revealed, as expected, that DRP, DNP, and DNR are more common in older individuals with longer diagnostic T2D, because of their association with disease progression. Significant difference in the levels of postprandial glucose was observed in the DRP and DNP cases when compared to the T2D group without complications. Higher levels of fasting glucose and HbA1c were found significant in the DNP group. These results suggest a relationship between the hyperglycemic status and the development of microvascular complications of T2D. Although higher levels of fasting glucose, postprandial glucose, and HbA1c were observed in patients with DNR, these levels were not significantly different from those found in T2D individuals without this complication. The small sample of individuals presenting DNR may explain the lack of significance concerning these variables in this group.

Patients with DRP and DNP showed lower BMI when compared to individuals without these complications. These findings seem contradictory, because obesity is considered a main risk factor for T2D and its complications [34]. This inconsistency may be explained by the fact that individuals with higher BMI were also those with shorter T2D onset and therefore presented fewer complications (data not shown). Curiously, a higher frequency of women was observed in the DNR group, although the reason underlying this observation is not clear. Studies involving a much larger sample may be necessary to confirm this finding and explore the possible causes for this observation.

DRP is the most frequent cause of newly detected cases of blindness in adults [1]. In the present study, DRP was associated with the GG genotype and G allele of TGF-*β*1 codon 25C/G polymorphism. This polymorphism is located in the region of the gene encoding the signal peptide and causes a change in the amino acid sequence (G/Arg → C/Pro) [35]. The G (Arg) allele has been associated with increased TGF-*β*1 production [36]. TGF-*β*1 modulates ocular cell migration and proliferation by inducing fibroblast growth factor-like and platelet-derived growth factor, which accelerate the process of retinal neovascularization [37]. TGF-*β*1 is also involved in extracellular matrix deposition (an essential step in new vessel formation) and stimulates angiogenesis in patients with ischemia and proliferative DRP [38]. Another case-control study with T2D patients with diabetic proliferative retinopathy found an association of the polymorphisms TGF-*β*1 codon 25C/G (G allele) and TGF-*β*1 codon 10T/C (T allele) with proliferative diabetic retinopathy [27]. On the contrary, a recent meta-analysis found an association between TGF-*β*1 codon 10T/C polymorphism and DRP [39]. Although the present study did not find significant association with other polymorphisms, the studies published by Paine et al. [18, 31] showed the association of the polymorphisms IL-10 -1082G/A and IFN-*γ* +874T/A with proliferative diabetic retinopathy. This discrepancy may be due to the genetic background of the populations studied since Paine et al. [18, 31] evaluated Indian ethnic groups.

DNP is a common cause of end-stage renal disease and the major cause of morbidity and premature mortality in patients with T2D [1]. Structurally, DNP is characterized by renal hypertrophy, mesangial matrix expansion, glomerulosclerosis, and tubulointerstitial fibrosis [40]. Recently, it has become evident that chronic inflammatory mechanisms contribute to the development and progression of DNP, such as infiltration of renal compartments by lymphocytes and monocytes (or macrophages) as well as local production of cytokines and chemokines in the kidney [41, 42]. Studies have observed that acute phase markers of inflammation (C reactive protein, fibrinogen, and IL-6) were correlated to proteinuria in T2D patients [43, 44] and showed that increased TNF-*α* levels were linked with DNP progression [45]. In our study, DNP was associated with lower frequency of GG genotype polymorphism IL-10 -1082G/A, which is related with higher expression of this cytokine. In fact, IL-10 is an anti-inflammatory cytokine and downregulates proinflammatory production of TNF-*α*, IL-6, and MCP-1 [46]. Thereby, lower production of IL-10 may be associated with a high production of proinflammatory cytokines and an exacerbated inflammatory response with subsequent renal injury in T2D patients. Previous studies revealed the association of other polymorphisms of IL-10 with DNP. Ezzidi et al. [26], in a case-control study with T2D patients from Tunisia, found a higher frequency of T allele (IL-10 -819T/C) in the group with DNP. Kung et al. [25] found higher frequency of the genotypes AA and CC for the IL-10 -592C/A polymorphism in individuals with the DNP in a Taiwanese population. In contrast to the current results, no association was found between the IL-10 -1082G/A polymorphism and DNP in patients from Turkey [47]. The polymorphism TGF-*β*1 codon 10T/C was associated with DNP risk in a meta-analysis conducted by Zhou et al. [29]. Thus, it is possible that the different polymorphisms may reflect the genetic background of the population studied.

IFN-*γ* is a pivotal proinflammatory cytokine implicated in the induction of immune mediated inflammatory response [48]. Studies suggest that IFN-*γ* participates in the pathogenesis of diabetes mellitus by upregulating the expression of MHC I/MHC II antigens and adhesion molecules on pancreatic *β* cells [49–51]. Herein we report an association of the AA genotype polymorphism IFN-*γ* +874T/A with lower glucose levels than those presented by patients carrying other genotypes. It is known that the allele +874T is associated with high IFN-*γ* levels, whereas the allele +874A is associated with low production of this cytokine [24]. Thus, decreased expression of IFN-*γ* may contribute to the downregulation inflammatory response in T2D patients and consequently to allowing better glycemic control.

It is known that obesity and visceral fat contribute to the development of hypertension, insulin resistance, and diabetes mellitus [34]. Typically, hypertension is a clinical condition commonly present in the patient at diagnosis of T2D, and the elevation of blood pressure often occurs before the onset of microalbuminuria [52]. In our study we found an association between the CC genotype and C allele for IL-10 -592C/A polymorphism and hypertension; furthermore we found a trend for association of the same polymorphism and BMI groups, since the -592AA genotype was less frequent in the obese T2D patients. In addition, we observed an association of the C allele for the IL-10 -819T/C polymorphism and patients with hypertension. For both IL-10 polymorphisms, the C allele is related with higher expression levels of this cytokine. Fichtlscherer et al. [53] reported that increased IL-10 levels were associated with improved systemic endothelial vasoreactivity in patients with elevated serum CRP levels, a condition commonly observed in T2D patients. Furthermore, Zeyda et al. [54] demonstrated that human adipose tissue macrophages (ATMs) produce high levels of IL-10. Consequently, increased levels of IL-10 could be associated with an exacerbated inflammatory profile in which the balance between pro- and anti-inflammatory cytokines contributes to chronic low-grade inflammation observed in obesity and T2D, as well as being involved in increased blood pressure.

Obesity also showed a relation to TGF-*β*1 codon 10T/C polymorphism. The CC genotype was more frequent in the group with BMI < 25 Kg/m^2^, while the TC genotype was more frequent in the group with BMI ≥ 30 Kg/m^2^. This polymorphism consists of a T → C transition at nucleotide 29 in the region encoding the signal peptide sequence, which results in a Leu → Pro substitution at amino acid 10. Studies have shown that the C allele increases the production of the TGF-*β*1 protein [55, 56]. The TGF-*β*1 is a multifunctional cytokine and shows anti-inflammatory action such as suppressing generation of free radicals, as well as vasculoprotective properties [57]. TGF-*β*1 can inhibit the adhesion and transmigration of neutrophils and T cells to the endothelium, inhibit production of adhesion molecules by the endothelial cells, and inhibit macrophage foam cell formation [58–61]. The higher frequency of the CC genotype found in patients with a BMI < 25 Kg/m^2^ suggests that the increased expression of TGF-*β*1 and its potential anti-inflammatory effect can facilitate the control of adiposity, since obese individuals have higher frequency TC genotype.

A higher frequency of T allele for the TGF-*β*1 codon 10T/C was observed in T2D patients with hypertension. Although some studies have demonstrated that TGF-*β*1 is associated with increased risk of essential hypertension through the stimulation of endothelin-1 expression in the vascular endothelium, release of renin from the juxtaglomerular cells in the kidney, and regulation of angiotensin II expression [62–64], no study has evaluated the impact of this polymorphism on hypertension in patients with diabetes. Thus, our results suggest that lower expression of TGF-*β*1 could predispose to diabetic hypertension due to lack of anti-inflammatory and protective TGF-*β*1 effects in vascular endothelium.

## 5. Conclusion

In conclusion, this is the first study to evaluate the association of a large panel of polymorphisms of cytokine genes with complications and comorbidities in T2D patients. However, the small sample of this study is considered a limitation and further studies including clinical classifications concerning nonproliferative/proliferative DRP and autonomic/chronic sensorimotor DNR may improve our current understanding about the link between the cytokine polymorphisms, their expression levels, and the development or progression of these complications. Although the results are not conclusive regarding the association of polymorphisms of cytokine genes with microvascular complications in T2D and comorbidities, taken together, our results may be relevant for future molecular studies aiming to predict possible T2D complications.

## Supplementary Material

Supplementary material shows in Table 1 the distribution of genotypes and alleles frequencies in T2D patients considering the complications: retinopathy, nephropathy and neuropathy. Table 2 presents the distribution of genotypes and alleles frequencies in T2D patients considering the comorbidities: hypertension, dyslipidemia, and body mass index (BMI).

## Figures and Tables

**Table 1:** Clinical and laboratorial characteristics in T2D patients considering retinopathy (DRP).

Variables	Diabetic retinopathy		
	Yes (*n* = 66)	No (*n* = 36)	*p*
Age (years)	57.00 (12.00)	52.50 (15.00)	0.013^∗^
Gender (male/female) %	22.73/77.27	11.11/88.89	0.150
BMI (Kg/m^2^)	29.95 ± 5.90	37.17 ± 6.95	<0.0001^∗^
Hypertension (yes/no) %	89.39/10.61	97.22/2.78	0.254
Dyslipidemia (yes/no) %	95.45/4.55	91.67/8.33	0.663
Fasting glucose (mg/dL)	143.00 (97.00)	118.00 (62.75)	0.074
Postprandial glucose (mg/dL)	225.00 (96.50)	144.50 (130.00)	0.013^∗^
HbA1c %	9.28 ± 1.62	8.31 ± 2.35	0.104
T2D onset (years) %			
≤5	7.58^b^	45.46	<0.0001^∗^
5–10	12.12	18.18	
≥10	80.30^a^	36.36	

BMI: body mass index and HbA1c: glycated hemoglobin.

Normal variables (BMI and HbA1c): the data are shown as mean ± standard deviation.

No normal variables (age, fasting glucose, and postprandial glucose): the data are shown as median (interquartile range).

Categorical variables (gender, hypertension, dyslipidemia, and T2D onset): the data are shown as “percentage of total.”

^∗^ *p* < 0.05 was considered statistically significant.

^a^More frequent; ^b^less frequent, residual analysis.

**Table 2:** Clinical and laboratorial characteristics in T2D patients considering nephropathy (DNP).

Variables	Diabetic nephropathy		
	Yes (*n* = 80)	No (*n* = 20)	*p*
Age (years)	58.00 (13.00)	50.50 (9.00)	<0.0001^∗^
Gender (male/female) %	21.25/78.75	10.00/90.00	0.348
BMI (Kg/m^2^)	30.48 ± 6.06	40.55 ± 5.23	<0.0001^∗^
Hypertension (yes/ no) %	90.00/10.00	100.00/0	0.204
Dyslipidemia (yes/no) %	96.25/3.75	85.00/15.00	0.092
Fasting glucose (mg/dL)	143.00 (97.50)	117.00 (55.50)	0.027^∗^
Postprandial glucose (mg/dL)	214.00 (107.50)	144.50 (122.25)	0.045^∗^
HbA1c %	9.24 ± 1.88	7.73 ± 1.84	0.006^∗^
T2D onset (years) %			
≤5	11.54^b^	47.37	0.001^∗^
5–10	14.10	15.79	
≥10	74.36^a^	36.84	

BMI: body mass index and HbA1c: glycated hemoglobin.

Normal variables (BMI and HbA1c): the data are shown as mean ± standard deviation.

No normal variables (age, fasting glucose, and postprandial glucose): the data are shown as median (interquartile range).

Categorical variables (gender, hypertension, dyslipidemia, and T2D onset): the data are shown as “percentage of total.”

^∗^ *p* < 0.05 was considered statistically significant.

^a^More frequent; ^b^less frequent, residual analysis.

**Table 3:** Clinical and laboratorial characteristics in T2D patients considering neuropathy (DNR).

Variables	Diabetic neuropathy		
	Yes (*n* = 42)	No (*n* = 60)	*p*
Age (years)	58.00 (12.00)	53.50 (15.00)	0.012^∗^
Gender (male/female) %	4.76/95.24	28.33/71.67	0.003^∗^
BMI (Kg/m^2^)	30.89 ± 5.79	33.84 ± 7.87	0.356
Hypertension (yes/no) %	90.48/9.52	93.33/6.67	0.714
Dyslipidemia (yes/no) %	100.00/0	90.00/10.00	0.079
Fasting glucose (mg/dL)	145.00 (95.00)	125.50 (68.00)	0.711
Postprandial glucose (mg/dL)	226.00 (100.00)	185.00 (107.25)	0.195
HbA1c %	9.22 ± 2.04	8.71 ± 1.90	0.969
T2D onset (years) %			
≤5	15.00	23.73	0.471
5–10	12.50	15.25	
≥10	72.50	61.02	

BMI: body mass index and HbA1c: glycated hemoglobin.

Normal variables (BMI and HbA1c): the data are shown as mean ± standard deviation.

No normal variables (age, fasting glucose, and postprandial glucose): the data are shown as median (interquartile range).

Categorical variables (gender, hypertension, dyslipidemia, and T2D onset): the data are shown as “percentage of total.”

^∗^ *p* < 0.05 was considered statistically significant.

**Table 4:** Haplotype distribution of IL-10 promoter gene (-1082G/A, -819T/C, and -512C/A) in T2D patients considering complications and comorbidities.

T2D complications/comorbidities		Haplotypes			*p* ^a^	*p* ^b^	*p* ^c^
		ACC%	ATA%	GCC%			
Retinopathy	Yes (*n* = 66)	31.07	34.08	34.08	0.778	0.563	0.778
	No (*n* = 36)	34.86	29.03	35.98			

Nephropathy	Yes (*n* = 80)	32.52	33.11	33.73	0.832	0.750	0.916
	No (*n* = 20)	32.58	29.92	37.42			

Neuropathy	Yes (*n* = 42)	29.77	38.08	30.94	0.670	0.300	0.550
	No (*n* = 60)	34.22	28.28	37.44			

Hypertension	Yes (*n* = 94)	34.06	30.30	35.62	0.418	0.204	0.418
	No (*n* = 8)	12.71	55.79	24.54			

Dyslipidemia	Yes (*n* = 96)	32.36	32.22	34.82	1.000	1.000	1.000
	No (*n* = 6)	34.02	32.46	32.54			

BMI	<30 (*n* = 44)	28.54	37.36	32.82	0.480	0.344	0.825
	≥30 (*n* = 58)	35.36	28.43	36.19			

BMI (body mass index): Kg/m^2^.

*p* < 0.05 was considered statistically significant.

*p* ^a^: ACC versus (ATA + GCC).

*p* ^b^: ATA versus (ACC + GCC).

*p* ^c^: GCC versus (ACC + ATA).
