# Supplementary material for: Association of a Large Panel of Cytokine Gene Polymorphisms with Complications and Comorbidities in Type 2 Diabetes Patients
Source: J Diabetes Res. 2015 May 6;2015:605965. doi: 10.1155/2015/605965 (PMC4438177; doi:10.1155/2015/605965)
Supplement: Supplementary file 1 — Supplementary material shows in Table 1 the distribution of genotypes and alleles frequencies in T2D patients considering the complications: retinopathy, nephropathy and neuropathy. Table 2 presents the distribution of genotypes and alleles frequencies in T2D patients considering the comorbidities: hypertension, dyslipidemia, and body mass index (BMI). [file 605965.f1.pdf]

SUPPLEMENTARY MATERIAL

Table SM1: Distributions of genotypes and alleles frequencies in T2D patients considering complications.

Polymorphisms		Diabetic Retinopathy				
		**Yes (n=66) (%)**	**No (n=36) (%)**	**p**	**OR**	**95% CI**
**TNF-α -308 A/G (rs 1800629)**	**Genotypes**					
	GG	49 (74.24)	29 (80.56)		Ref.	
	GA	17 (25.76)	6 (16.67)	0.252	1.677	0.594 - 4.734
	AA	0	1 (2.77)		-	-
	**Alleles**					
	G	115 (87.12)	64 (88.89)	0.713	0.846	0.314 - 2.224
	A	17 (12.88)	8 (11.11)			
**TGF-β1 codon 10 T/C (rs 1982073)**	**Genotypes**					
	TT	25 (37.88)	10 (27.78)		Ref.	
	TC	31 (46.97)	23 (63.89)	0.246	1.855	0.746 - 4.610
	CC	10 (15.15)	3 (8.33)		0.750	0.170 - 3.308
	**Alleles**					
	T	81 (61.36)	43 (59.72)	0.819	1.071	0.571 - 2.008
	C	51 (38.64)	29 (40.28)			

**TGF-β1 codon 25 C/G (rs 1800471)**	**Genotypes**					
	GG	61 (92.42)	26 (72.22)		Ref.	
	GC	4 (6.06)	10 (27.78)	0.004*	5.865	1.685 - 20.413
	CC	1 (1.52)	0,0		-	-
	**Alleles**					
	G	126 (95.45)	62 (86.11)	0.018*	3.387	1.069 - 11.064
	C	6 (4.55)	10 (13.89)			
**IL-10 -1082 G/A (rs 1800896)**	**Genotypes**					
	GG	8 (12.12)	4 (11.11)		Ref.	
	GA	29 (43.94)	18 (50.00)	0.841	1.036	0.266 - 4.032
	AA	29 (43.94)	14 (38.89)		1.286	0.540 - 3.062
	**Alleles**					
	G	45 (34.09)	26 (36.11)	0.772	0.915	0.418 - 1.744
	A	87 (65.91)	46 (63.89)			
**IL-10 -819 C/T (rs 1800871)**	**Genotypes**					
	TT	10 (15.15)	3 (8.33)		Ref.	
	TC	25 (37.88)	15 (41.67)	0.613	2.000	0.474 - 8.444
	CC	31 (46.97)	18 (50.00)		1.935	0.470 - 7.967
	**Alleles**					
	T	45 (34.09)	21 (29.17)	0.472	1.256	0.645 - 2.457

	C	87 (65.91)	51 (70.83)			
**IL-10 -592 C/A (rs 1800872)**	**Genotypes**					
	CC	31 (46.97)	18 (50.00)		Ref.	
	CA	24 (36.36)	15 (41.67)	0.498	2.129	0.524 - 8.655
	AA	11 (16.67)	3 (8.33)		2.292	0.548 - 9.579
	**Alleles**					
	C	86 (65.15)	51 (70.83)	0.409	0.770	0.394 - 1.498
	A	46 (34.85)	21 (29.17)			
**IL-6 -174 C/G (rs 1800795)**	**Genotypes**					
	GG	46 (69.69)	23 (63.89)		Ref.	
	GC	15 (22.73)	11 (30.56)	0.692	1.467	0.582 - 3.699
	CC	5 (7.58)	2 (5.55)		0.800	0.144 - 4.443
	**Alleles**					
	G	107 (81.06)	57 (79.17)	0.745	1.126	0.517 - 2.438
	C	25 (18.94)	15 (20.83)			
**INF-γ +874 T/A (rs 2430561)**	**Genotypes**					
	TT	10 (15.15)	3 (8.33)		Ref.	
	TA	30 (45.45)	15 (41.67)	0.465	0.433	0.104 - 1.799
	AA	26 (39.39)	18 (50.00)		0.722	0.305 - 1.712
	**Alleles**					

	T	50 (37.88)	21 (29.17)	0.212	1.481	0.764 - 2.882
	A	82 (62.12)	51 (70.83)			

**Polymorphisms**		**Diabetic Nephropathy**				
		**Yes (n=80) (%)**	**No (n=20) (%)**	**p**	**OR**	**CI (95%)**
**TNF-α -308 A/G (rs 1800629)**	**Genotypes**					
	GG	61 (76.25)	16 (80.00)	0.164	Ref.	
	GA	19 (23.75)	3 (15.00)		1.661	0.437 - 6.321
	AA	0	1 (5.00)		-	-
	**Alleles**					
	G	141 (88.13)	35 (87.50)	1.000	1.060	0.321 - 3.297
	A	19 (11.87)	5 (12.50)			
**TGF-β1 codon 10 T/C (rs 1982073)**	**Genotypes**					
	TT	28 (35.00)	6 (30.00)	0.363	Ref.	
	TC	40 (50.00)	13 (65.00)		1.517	0.514 - 4.471
	CC	12 (15.00)	1 (5.00)		0.389	0.042 - 3.589
	**Alleles**					
	T	96 (60.00)	25 (62.50)	0.772	0.900	0.414 - 1.942
	C	64 (40.00)	15 (37.50)			
**TGF-β1 codon 25 C/G**	**Genotypes**					

(rs 1800471)	GG	69 (86.25)	16 (80.00)		Ref.	
	GC	10 (12.50)	4 (20.00)	0.578	1.725	0.479 - 6.209
	CC	1 (1.25)	0		-	-
	**Alleles**					
	G	148 (92.50)	36 (90.00)	0.532	1.370	0.349 - 4.962
	C	12 (7.50)	4 (10.00)			
**IL-10 -1082 G/A (rs 1800896)**	**Genotypes**					
	GG	7 (8.75)	5 (25.00)		Ref.	
	GA	40 (50.00)	5 (25.00)	0.049*	2.357	1.612 - 9.076
	AA	33 (41.25)	10 (50.00)		0.413	1.128 - 1.327
	**Alleles**					
	G	54 (33.75)	15 (37.50)	0.655	0.849	0.391 - 1.854
	A	106 (66.25)	25 (62.50)			
**IL-10 -819 C/T (rs 1800871)**	**Genotypes**					
	TT	11 (13.75)	2 (10.00)		Ref.	
	TC	31 (38.75)	8 (40.00)	0.905	1.419	0.261 - 7.733
	CC	38 (47.50)	10 (50.00)		1.447	0.275 - 7.611
	**Alleles**					
	T	53 (33.13)	12 (30.00)	0.706	1.156	0.514 - 2.628
	C	107 (66.87)	28 (70.00)			
**IL-10 -592 C/A**	**Genotypes**					

**(rs 1800872)**	CC	38 (47.50)	10 (50.00)		Ref.	
	CA	30 (37.50)	8 (40.00)	0.847	1.579	0.303 - 8.230
	AA	12 (15.00)	2 (10.00)		1.600	0.296 - 8.653
	**Alleles**					
	C	106 (66.25)	28 (70.00)	0.652	0.841	0.370 - 1.889
	A	54 (33.75)	12 (30.00)			
**IL-6 -174 C/G (rs 1800795)**	**Genotypes**					
	GG	55 (68.75)	13 (65.00)		Ref.	
	GC	19 (23.75)	6 (30.00)	0.808	1.336	0.445 - 4.010
	CC	6 (7.50)	1 (5.00)		0.705	0.078 - 6.374
	**Alleles**					
	G	129 (80.63)	32 (80.00)	0.929	1.040	0.397 - 2.653
	C	31 (19.37)	8 (20.00)			
**INF-γ +874 T/A (rs 2430561)**	**Genotypes**					
	TT	10 (12.50)	3 (15.00)		Ref.	
	TA	38 (47.50)	6 (30.00)	0.363	0.873	0.203 - 3.761
	AA	32 (40.00)	11 (55.00)		0.459	0.153 - 1.380
	**Alleles**					
	T	58 (36.25)	12 (30.00)	0.459	1.327	0.593 - 3.007
	A	102 (63.75)	28 (70.00)			

Polymorphisms		Diabetic Neuropathy				
		Yes (n=42) (%)	No (n=60) (%)	p	OR	CI (95%)
**TNF-α -308 A/G (rs 1800629)**	**Genotypes**					
	GG	36 (85.71)	42 (70.00)		Ref.	
	GA	6 (14.29)	17 (28.33)	0.118	0.412	0.147 - 1.155
	AA	0	1 (1.67)		-	-
	**Alleles**					
	G	78 (92.86)	101 (84.17)	0.062	2.446	0.868 - 7.221
	A	6 (7.14)	19 (15.83)			
**TGF-β1 codon 10 T/C (rs 1982073)**	**Genotypes**					
	TT	13 (30.95)	22 (36.67)		Ref.	
	TC	23 (54.76)	31 (51.67)	0.814	0.796	0.333 - 1.906
	CC	6 (14.29)	7 (11.66)		0.689	0.190 - 2.500
	**Alleles**					
	T	49 (58.33)	75 (62.50)	0.549	0.840	0.456 - 1.546
	C	35 (41.67)	45 (37.50)			
**TGF-β1 codon 25 C/G (rs 1800471)**	**Genotypes**					
	GG	34 (81.00)	53 (88.33)		Ref.	
	GC	8 (19.00)	6 (10.00)	0.307	0.481	0.153 - 1.509
	CC	0	1 (1.67)		-	-

	**Alleles**					
	G	76 (90.48)	112 (93.33)	0.455	0.679	0.220 - 2.095
	C	8 (9.52)	8 (6.67)			
**IL-10 -1082 G/A (rs 1800896)**	**Genotypes**					
	GG	3 (7.14)	9 (15.00)		Ref.	
	GA	20 (47.62)	27 (45.00)	0.474	2.375	0.563 - 10.010
	AA	19 (45.24)	24 (40.00)		1.069	0.464 - 2.462
	**Alleles**					
	G	26 (30.95)	45 (37.50)	0.334	0.747	0.396 - 1.408
	A	58 (69.05)	75 (62.50)			
**IL-10 -819 C/T (rs 1800871)**	**Genotypes**					
	TT	8 (19.05)	5 (8.33)		Ref.	
	TC	16 (38.10)	24 (40.00)	0.266	2.400	0.665 - 8.666
	CC	18 (42.85)	31 (51.67)		2.756	0.782 - 9.708
	**Alleles**					
	T	32 (38.10)	34 (28.33)	0.142	1.557	0.824 - 2.941
	C	52 (61.90)	86 (71.67)			
**IL-10 -592 C/A (rs 1800872)**	**Genotypes**					
	CC	18 (42.86)	31 (51.67)		Ref.	
	CA	15 (35.71)	24 (40.00)	0.165	3.100	0.899 - 10.690
	AA	9 (21.43)	5 (8.33)		2.880	0.809 - 10.249

	**Alleles**					
	C	51 (60.71)	86 (71.67)	0.101	0.611	0.324 - 1.151
	A	33 (39.29)	34 (28.33)			
**IL-6 -174 C/G (rs 1800795)**	**Genotypes**					
	GG	27 (64.29)	42 (70.00)	0.891	Ref.	
	GC	12 (28.57)	14 (23.33)		0.857	0.178 - 4.133
	CC	3 (7.14)	4 (6.67)		0.750	0.302 - 1.863
	**Alleles**					
	G	66 (78.57)	98 (81.67)	0.584	0.823	0.388 - 1.749
	C	18 (21.43)	22 (18.33)			
**INF-γ +874 T/A (rs 2430561)**	**Genotypes**					
	TT	4 (9.52)	9 (15.00)	0.455	Ref.	
	TA	17 (40.48)	28 (46.67)		2.054	0.550 - 7.675
	AA	21 (50.00)	23 (38.33)		1.504	0.646 - 3.499
	**Alleles**					
	T	25 (29.76)	46 (38.33)	0.206	0.682	0.360 - 1.288
	A	59 (70.24)	74 (61.67)			

*p<0.05 was considered statistically significant.
Ref. = reference

Table SM2: Distributions of genotypes and alleles frequencies in T2D patients considering comorbidities.

Polymorphisms		Hypertension				
		Yes (n=94) (%)	No (n=8) (%)	p	OR	CI (95%)
**TNF-α -308 A/G (rs 1800629)**	**Genotypes**					
	GG	71 (75.53)	7 (87.50)	0.704	Ref.	
	GA	22 (23.40)	1 (12.50)		0.461	0.054 - 3.955
	AA	1 (1.07)	0		-	-
	**Alleles**					
	G	164 (87.23)	15 (93.75)	0.699	0.456	0.021 - 3.558
	A	24 (12.77)	1 (6.25)			
**TGF-β1 codon 10 T/C (rs 1982073)**	**Genotypes**					
	TT	34 (36.17)	1 (12.50)	0.073	Ref.	
	TC	50 (53.19)	4 (50.00)		0.368	0.039 - 3.433
	CC	10 (10.64)	3 (37.50)		0.098	0.009 - 1.049
	**Alleles**					
	T	118 (62.77)	6 (37.50)	0.047*	2.810	1.890 - 9.148
	C	70 (37.23)	10 (62.50)			
**TGF-β1 codon 25 C/G (rs 1800471)**	**Genotypes**					
	GG	80 (85.11)	7 (87.50)	1.000	Ref.	
	GC	13 (13.83)	1 (12.50)		1.137	0.129 - 10.020

	CC	1 (1.06)	0		-	-
	**Alleles**					
	G	173 (92.02)	15 (93.75)	1.000	0.769	0.036 - 6.265
	C	15 (7.98)	1 (6.25)			
**IL-10 -1082 G/A (rs 1800896)**	**Genotypes**					
	GG	12 (12.77)	0		-	-
	GA	43 (45.74)	4 (50.00)	0.694	1.103	0.258 - 4.710
	AA	39 (41.49)	4 (50.00)		Ref.	
	**Alleles**					
	G	67 (35.64)	4 (25.00)	0.586	1.661	0.472 - 6.383
	A	121 (64.36)	12 (75.00)			
**IL-10 -819 C/T (rs 1800871)**	**Genotypes**					
	TT	10 (10.64)	3 (37.50)		Ref.	
	TC	37 (39.36)	3 (37.50)	0.079	3.700	0.645 - 21.210
	CC	47 (50.00)	2 (25.00)		7.050	1.039 - 47.846
	**Alleles**					
	T	57 (30.32)	9 (56.25)	0.033*	0.338	1.107 - 1.154
	C	131 (69.68)	7 (43.75)			
**IL-10 -592 C/A (rs 1800872)**	**Genotypes**					
	CC	47 (50.00)	2 (25.00)	0.013*	Ref.	
	CA	37 (39.36)	2 (25.00)		9.400	1.509 - 58.568

	AA	10 (10.64)	4 (50.00)		7.400	1.181 - 46.385
	**Alleles**					
	C	131 (69.68)	6 (37.50)	0.009*	3.830	1.206 - 12.546
	A	57 (30.32)	10 (62.50)			
**IL-6 -174 C/G (rs 1800795)**	**Genotypes**					
	GG	65 (69.15)	4 (50.00)	0.117	Ref.	
	GC	24 (25.53)	2 (25.00)		0.738	0.127 - 4.295
	CC	5 (5.32)	2 (25.00)		0.154	0.022 - 1.055
	**Alleles**					
	G	154 (81.91)	10 (62.50)	0.06	2.718	0.811 - 8.878
	C	34 (18.09)	6 (37.50)			
**INF-γ +874 T/A (rs 2430561)**	**Genotypes**					
	TT	11 (11.70)	2 (25.00)	0.236	Ref.	
	TA	40 (42.55)	5 (62.50)		0.128	0.011 - 1.543
	AA	43 (45.74)	1 (12.50)		0.186	0.021 - 1.662
	**Alleles**					
	T	62 (32.98)	9 (56.25)	0.061	0.383	0.121 - 1.189
	A	126 (67.02)	7 (43.75)			

Polymorphisms		Dyslipidemia				
		Yes (n=96) (%)	No (n=6) (%)	p	OR	CI (95%)
**TNF-α -308 A/G (rs 1800629)**	**Genotypes**					
	GG	73 (76.04)	5 (83.33)		Ref.	
	GA	22 (22.92)	1 (16.67)	1.000	1.507	0.167 - 13.590
	AA	1 (1.04)	0		-	-
	**Alleles**					
	G	168 (87.50)	11 (91.67)	1.000	0.636	0.029 - 5.160
	A	24 (12.50)	1 (8.33)			
**TGF-β1 codon 10 T/C (rs 1982073)**	**Genotypes**					
	TT	34 (35.42)	1 (16.67)		Ref.	
	TC	50 (52.08)	4 (66.66)	0.858	2.720	0.291 - 25.402
	CC	12 (12.50)	1 (16.67)		2.833	0.164 - 48.926
	**Alleles**					
	T	118 (61.46)	6 (50.00)	0.43	1.595	0.435 - 5.847
	C	74 (38.54)	6 (50.00)			
**TGF-β1 codon 25 C/G (rs 1800471)**	**Genotypes**					
	GG	82 (85.42)	5 (83.33)		Ref.	
	GC	13 (13.54)	1 (16.67)	1.000	1.262	0.136 - 11.677
	CC	1 (1.04)	0		-	-

	**Alleles**					
	G	177 (92.19)	11 (91.67)	1.000	1.073	0.049 - 9.092
	C	15 (7.81)	1 (8.33)			
**IL-10 -1082 G/A (rs 1800896)**	**Genotypes**					
	GG	11 (11.46)	1 (16.67)	0.87	Ref.	
	GA	45 (46.88)	2 (33.33)		1.212	0.115 - 12.831
	AA	40 (41.66)	3 (50.00)		0.593	0.094 - 3.728
	**Alleles**					
	G	67 (34.90)	4 (33.33)	1.000	1.047	0.272 - 4.310
	A	125 (65.10)	8 (66.67)			
**IL-10 -819 C/T (rs 1800871)**	**Genotypes**					
	TT	12 (12.50)	1 (16.67)	1.000	Ref.	
	TC	38 (39.58)	2 (33.33)		0.632	0.053 - 7.592
	CC	46 (47.92)	3 (50.00)		0.783	0.075 - 8.211
	**Alleles**					
	T	62 (32.29)	4 (33.33)	1.000	0.954	0.248 - 3.936
	C	130 (67.71)	8 (66.67)			
**IL-10 -592 C/A (rs 1800872)**	**Genotypes**					
	CC	46 (47.92)	3 (50.00)	1.000	Ref.	
	CA	37 (38.54)	2 (33.33)		0.848	0.081 - 8.849
	AA	13 (13.54)	1 (16.67)		0.703	0.059 - 8.410

	**Alleles**					
	C	129 (67.19)	8 (66.67)	1.000	1.024	0.248 - 3.942
	A	63 (32.81)	4 (33.33)			
**IL-6 -174 C/G (rs 1800795)**	**Genotypes**					
	GG	66 (68.75)	3 (50.00)	0.457	Ref.	
	GC	24 (25.00)	2 (33.33)		1.833	0.289 - 11.650
	CC	6 (6.25)	1 (16.67)		3.667	0.328 - 40.929
	**Alleles**					
	G	156 (81.25)	8 (66.67)	0.257	2.167	0.515 - 8.552
	C	36 (18.75)	4 (33.33)			
**INF-γ +874 T/A (rs 2430561)**	**Genotypes**					
	TT	12 (12.50)	1 (16.67)	0.336	Ref.	
	TA	44 (45.83)	1 (16.67)		0.833	0.085 - 8.182
	AA	40 (41.67)	4 (66.66)		0.227	0.024 - 2.119
	**Alleles**					
	T	68 (35.42)	3 (25.00)	0.548	1.645	0.390 - 7.956
	A	124 (64.58)	9 (75.00)			

Polymorphisms		BMI					
		< 25 Kg/m^2 (n=19) (%)	25 - 30 Kg/m^2 (n=25) (%)	≥ 30 Kg/m^2 (n=58) (%)	p	OR[#]	CI (95%)
**TNF-α -308 A/G (rs 1800629)**	**Genotypes**						
	GG	14 (73.68)	22 (88.00)	42 (72.41)		Ref. (BMI < 25 Kg/m^2)	
	GA	5 (26.32)	3 (12.00)	15 (25.86)	0.625	0.838	0.638 - 1.100
	AA	0	0	1 (1.73)		-	-
	**Alleles**						
	G	33 (86.84)	47 (94.00)	99 (85.34)	p^a,p^b,p^c>0.05	0.421	0.073 - 2.227
	A	5 (13.16)	3 (6.00)	17 (14.66)		1.133	0.355 - 3.831
**TGF-β1 codon 10 T/C (rs 1982073)**	**Genotypes**						
	TT	5 (26.32)	12 (48.00)	18 (31.04)		Ref. (BMI < 25 Kg/m^2)	
	TC	8 (42.10)	10 (40.00)	36 (62.07)[a]	0.026*	1.963	1.007 - 3.826
	CC	6 (31.58)[a]	3 (12.00)	4 (6.89)		1.546	1.858 - 2.786
	**Alleles**						
	T	18 (47.37)	34 (68.00)	72 (62.07)	p^a,p^b,p^c>0.05	0.424	0.161 - 1.104
	C	20 (52.63)	16 (32.00)	44 (37.93)		0.550	0.246 - 1.226
**TGF-β1 codon 25 C/G (rs 1800471)**	**Genotypes**						
	GG	16 (84.21)	21 (84.00)	50 (86.21)		Ref. (BMI < 25 Kg/m^2)	
	GC	3 (15.79)	3 (12.00)	8 (13.79)	0.613	0.562	0.169 - 1.870
	CC	0	1 (4.00)	0		0.579	0.189 - 1.778

	**Alleles**						
	G	35 (92.11)	45 (90.00)	108 (93.10)	$p^a,p^b,p^c>0.05$	1.296	0.245 - 7.439
	C	3 (7.89)	5 (10.00)	8 (6.90)		0.864	0.193 - 4.375
**IL-10 -1082 G/A (rs 1800896)**	**Genotypes**						
	GG	2 (10.53)	2 (8.00)	8 (13.79)	0.677	Ref. (BMI < 25 Kg/m^2)	
	GA	11 (57.89)	10 (40.00)	26 (44.83)		0.902	0.697 - 1.168
	AA	6 (31.58)	13 (52.00)	24 (41.38)		0.712	0.558 - 0.908
	**Alleles**						
	G	15 (39.47)	14 (28.00)	42 (36.21)	$p^a,p^b,p^c>0.05$	1.677	0.623 - 4.533
	A	23 (60.53)	36 (72.00)	74 (63.79)		1.149	0.506 - 2.600
**IL-10 -819 C/T (rs 1800871)**	**Genotypes**						
	TT	4 (21.05)	5 (20.00)	4 (6.90)	0.133	Ref. (BMI < 25 Kg/m^2)	
	TC	9 (47.37)	6 (24.00)	25 (43.10)		0.870	0.672 - 1.125
	CC	6 (31.58)	14 (56.00)	29 (50.00)		0.622	0.484 - 0.799
	**Alleles**						
	T	17 (44.74)	16 (32.00)	33 (28.45)	$p^a,p^b,p^c>0.05$	1.720	0.657 - 4.524
	C	21 (55.26)	34 (68.00)	83 (71.55)		2.036	0.894 - 4.639
**IL-10 -592 C/A (rs 1800872)**	**Genotypes**						
	CC	6 (31.58)	14 (56.00)	29 (50.00)	0.058	Ref. (BMI < 25 Kg/m^2)	
	CA	9 (47.37)	5 (20.00)	25 (43.10)		0.876	0.677 - 1.134
	AA	4 (21.05)	6 (24.00)	4 (6.90)		0.621	0.483 - 0.798

	**Alleles**						
	C	21 (55.26)	33 (66.00)	83 (71.55)	$p^a, p^b, p^c > 0.05$	0.636	0.244 - 1.654
	A	17 (44.74)	17 (34.00)	33 (28.45)		0.461	0.216 - 1.118
**IL-6 -174 C/G (rs 1800795)**	**Genotypes**						
	GG	13 (68.42)	15 (60.00)	41 (70.69)		Ref. (BMI < 25 Kg/m^2)	
	GC	5 (26.32)	7 (28.00)	14 (24.14)	0.806	0.784	0.594 - 1.034
	CC	1 (5.26)	3 (12.00)	3 (5.17)		0.661	0.510 - 0.856
	**Alleles**						
	G	31 (81.58)	37 (74.00)	96 (82.76)	$p^a, p^b, p^c > 0.05$	1.556	0.467 - 4.977
	C	7 (18.42)	13 (26.00)	20 (17.24)		0.923	0.328 - 2.669
**INF-γ +874 T/A (rs 2430561)**	**Genotypes**						
	TT	3 (15.78)	4 (16.00)	6 (10.34)		Ref. (BMI < 25 Kg/m^2)	
	TA	8 (42.11)	15 (60.00)	22 (37.93)	0.228	0.737	0.393 - 1.384
	AA	8 (42.11)	6 (24.00)	30 (51.73)		1.251	0.701 - 2.232
	**Alleles**						
	T	14 (36.84)	23 (46.00)	34 (29.31)	$p^c = 0.038$* $p^a, p^b > 0.05$	0.685	1.264 - 1.769
	A	24 (63.16)	27 (54.00)	82 (70.69)		1.407	0.606 - 3.250

BMI - Body Mass Index
*p<0.05 was considered statistically significant
[#]BMI < 25 Kg/m^2 was reference in the Odds Ratio estimation for genotype and allele frequencies - BMI < 25 Kg/m^2 *versus* BMI 25 - 30 Kg/m^2 and BMI < 25 Kg/m^2 *versus* BMI ≥ 30 Kg/m^2, respectively

p^a: group (BMI < 25 Kg/m^2) *versus* group (BMI 25 - 30 Kg/m^2)
p^b: group (BMI < 25 Kg/m^2) *versus* group (BMI ≥ 30 Kg/m^2)

p^c: group (BMI 25 - 30 Kg/m^2) *versus* group (BMI ≥ 30 Kg/m^2)
a (more frequent) – residual analysis
